# Hydrogen Sulfide Attenuates High-Fat Diet-Induced Non-Alcoholic Fatty Liver Disease by Inhibiting Apoptosis and Promoting Autophagy via Reactive Oxygen Species/Phosphatidylinositol 3-Kinase/AKT/Mammalian Target of Rapamycin Signaling Pathway

**DOI:** 10.3389/fphar.2020.585860

**Published:** 2020-11-30

**Authors:** Dongdong Wu, Peiyu Zhong, Yizhen Wang, Qianqian Zhang, Jianmei Li, Zhengguo Liu, Ailing Ji, Yanzhang Li

**Affiliations:** ^1^ School of Basic Medical Sciences, Henan University, Kaifeng, China; ^2^ Henan International Joint Laboratory for Nuclear Protein Regulation, Henan University, Kaifeng, China; ^3^ School of Stomatology, Henan University, Kaifeng, China

**Keywords:** hydrogen sulfide, nonalcoholic fatty liver disease, apoptosis, autophagy, signaling pathway

## Abstract

Non-alcoholic fatty liver disease (NAFLD) is a common chronic liver disease worldwide. Hydrogen sulfide (H_2_S) is involved in a wide range of physiological and pathological processes. Nevertheless, the mechanism of action of H_2_S in NAFLD development has not been fully clarified. Here, the reduced level of H_2_S was observed in liver cells treated with oleic acid (OA). Administration of H_2_S increased the proliferation of OA-treated cells. The results showed that H_2_S decreased apoptosis and promoted autophagy through reactive oxygen species (ROS)-mediated phosphatidylinositol 3-kinase (PI3K)/AKT/mammalian target of rapamycin (mTOR) cascade in OA-treated cells. In addition, administration of H_2_S relieved high-fat diet (HFD)-induced NAFLD via inhibition of apoptosis and promotion of autophagy. These findings suggest that H_2_S could ameliorate HFD-induced NAFLD by regulating apoptosis and autophagy through ROS/PI3K/AKT/mTOR signaling pathway. Novel H_2_S-releasing donors may have therapeutic potential for the treatment of NAFLD.

## Introduction

Hydrogen sulfide (H_2_S) is one of the gaseous transmitters in organisms (Wang, 2012; Hartle and Pluth, 2016; Szabo, 2016). H_2_S can be produced from homocysteine and l-cysteine (L-Cys) through the catalytic action of cystathionine β-synthase (CBS) and cystathionine γ-lyase (CSE). CSE and CBS are mainly detected in cytosol (Wang, 2012; Hartle and Pluth, 2016). In addition, 3-mercaptopyruvate sulfurtransferase (3-MST) is a member of the pyridoxal-5′-phosphate-independent enzymes. In the presence of α-ketoglutarate, 3-MST can act in combination with cysteine aminotransferase (CAT) to produce H_2_S from L-Cys. 3-MST and CAT have shown both cytosolic and mitochondrial localizations (Wang, 2012; Szabo, 2016). Furthermore, d-amino acid oxidase can metabolize d-cysteine to 3-mercaptopyruvate, which serves as a substrate for 3-MST to produce H_2_S in kidney and brain (Shibuya et al., 2013). H_2_S could be rapidly stored or released in two main forms such as acid-labile sulfide and bound sulfane sulfur in cells (Shen et al., 2013; Wu et al., 2018).

H_2_S is essential in different types of physiological processes, such as angiogenesis (Longchamp et al., 2018), vascular relaxation (Cheng et al., 2018), and energy production (Fu et al., 2012). The abnormal metabolism of H_2_S can lead to many types of diseases, including diabetes (Cheng et al., 2016), atherosclerosis (Mani et al., 2013), and neurodegenerative diseases (Paul et al., 2014). Non-alcoholic fatty liver disease (NAFLD) has emerged as an important cause of chronic liver disease worldwide (Rinella and Sanyal, 2016; Santhekaduret al., 2018; Targheret al., 2018). NAFLD is caused by the build-up of lipids in the liver, which can increase the risks of hepatocellular carcinoma and end-stage liver diseases (Rinella and Sanyal, 2016; Petta et al., 2018). Many risk factors are involved in the development of NAFLD, including diabetes, obesity, hyperlipidemia, and certain medications (Rodriguezet al., 2012; Santhekaduret al., 2018). It has been shown that H_2_S mitigates the fatty liver through the promotion of antioxidant potential and lipid metabolism in obese mice (Wu et al., 2015). Nonetheless, the mechanism of action of H_2_S in the progression of NAFLD remains to be further elucidated.

In the current study, the levels of endogenous H_2_S in oleic acid (OA)-treated liver cells were examined and the roles of exogenous H_2_S in the proliferation, apoptosis, and autophagy of OA-treated liver cells were determined. Furthermore, a mouse model of high-fat diet (HFD)-induced NAFLD was used to confirm the mechanism of action of exogenous H_2_S in NAFLD.

## Materials and Methods

### Cell Culture

Human liver cell lines QSG-7701 and L02 were obtained from Jiangsu Feiya Biological Technology Co., Ltd. (Yancheng, Jiangsu, China), and cultured in DMEM, 10% fetal bovine serum, streptomycin (100 μg/ml), and penicillin (100 U/ml). A recent study indicates that exposure of HepG2 cells to free fatty acids or high glucose results in a significant increase in intracellular lipids, while co-incubation with 30 μM NaHS (an H_2_S donor) for 72 h reduces acetyl-CoA contents and lipid accumulation. Furthermore, blockage of CSE activity could promote intracellular lipid accumulation (Ali et al., 2020). In the present study, liver cells were incubated in the medium containing 0.5 mM bovine serum albumin (BSA)-OA complex (1:4, molar ratio), with or without 100 µM NaHS (Sigma, Shanghai, China) for 24 h. The control group was treated with BSA.

### Measurement of Hydrogen Sulfide Concentration

H_2_S concentrations in the culture supernatant and liver cells were detected using enzyme-linked immunosorbent assay (ELISA) kits (LanpaiBio, Shanghai, China).

### Oil Red O Staining

Cells were fixed with 4% paraformaldehyde for 30 min and incubated with 60% isopropanol for 15 min. Cells were incubated with Oil Red O (ORO) staining solution for 20 min, then counterstained with hematoxylin. The intensity was calculated using Image J software (National Institutes of Health, Bethesda, MD, United States) (Qiu et al., 2018).

### Cell Growth Assay

The 5-ethynyl-2′-deoxyuridine (EdU) assay kits (RiboBio, Guangzhou, Guangdong, China) were adopted to detect cell proliferation. The proliferation rate was calculated as the ratio of positive cells to total cells (Wu et al., 2019a). 3-(4,5)-dimethylthiahiazo (-z-y1)-3,5-di-phenytetrazoliumromide (MTT) (Sigma, Shanghai, China) assay was further adopted. The cell viability was calculated as a ratio to the control group (Wu et al., 2019b).

### Flow Cytometry

Cells were trypsinized, washed with phosphate buffered saline (PBS), then fixed in 75% ethanol (ice-cold) overnight at 4°C. Cells were incubated in RNase A/propidium iodide (PI) mixture at room temperature for 30 min. Cell cycle distribution was determined by a flow cytometer (FACSVerse, BD Biosciences, San Jose, CA, United States). The apoptotic levels were detected by Annexin V-FITC apoptosis detection kits (Beyotime, Haimen, Jiangsu, China) and analyzed using the flow cytometer.

### Terminal Deoxynucleotidyl Transferase-Mediated dUTP-Biotin Nick End Labeling Assay

Apoptosis was evaluated using the *in situ* cell death detection kits (Beyotime, Haimen, Jiangsu, China). The apoptotic index was calculated as the percentage of Terminal deoxynucleotidyl transferase dUTP nick end labeling (TUNEL) positive cells to the total number of cells (Zhang et al., 2018a).

### Immunofluorescence Staining

The green fluorescent protein (GFP)-red fluorescent protein (RFP)-LC3 plasmid is widely adopted to determine the autophagic level (Lee et al., 2017). The plasmid (Hanbio Biotechnology, Shanghai, China) was transfected into liver cells for 48 h. The fluorescence was then visualized by a fluorescent microscope (Eclipse Ti, Nikon, Melville, NY, United States). The auto-lysosomes (free red dots) and autophagosomes (yellow dots) were counted as the percentage of positive cells to total cells (Wang et al., 2016).

### Determination of Reactive Oxygen Species (ROS)

The levels of Reactive Oxygen Species (ROS) were measured by ROS detection assay kits (Beyotime, Haimen, Jiangsu, China).

### Antioxidant Activity Analysis

The activities of superoxide dismutase (SOD), catalase (CAT), and glutathione peroxidase (GSH-Px) were detected by commercial kits (Beyotime, Haimen, Jiangsu, China).

### Western Blot Assay

Western blot assay was carried out as previously described (Wang et al., 2019). The primary antibodies, including anti-CBS, anti-CSE, anti-3-MST, anti-cyclin E1, anti-cyclin D1, anti-cyclin-dependent kinase-2 (CDK2), anti-cyclin-dependent kinase-4 (CDK4), anti-p27, anti-p21, anti-beclin-1, anti-LC3A/B, anti-P62, anti-phosphatidylinositol 3-kinase (PI3K), anti-AKT, anti-mammalian target of rapamycin (mTOR), anti-phospho (p)-PI3K (Tyr199/Tyr458), anti-p-AKT (Ser473), and anti-p-mTOR (Ser2448) were purchased from Cell Signaling Technology (CST, Danvers, MA, United States). The primary antibodies, including anti-cleaved caspase (cas)-3, anti-cleaved cas-9, anti-cleaved poly adenosine diphosphate-ribose polymerase (PARP), anti-β-actin, and the horseradish peroxidase-conjugated secondary antibody were purchased from Proteintech (Chicago, IL, United States). The immunoreactive bands were visualized by a chemiluminescence detection system (Thermo Fisher, Waltham, MA, United States).

### Animals

All animal experiments were approved by the Committee of Medical Ethics and Welfare for Experimental Animals of Henan University School of Medicine (HUSOM-2017-192). Eighteen male C57BL/6J mice (8-week-old), low-fat diet (LFD, 10% kcal fat, 20% kcal protein, and 70% kcal carbohydrate), and HFD (45% kcal fat, 20% kcal protein, and 35% kcal carbohydrate) were purchased from Vital River (Beijing, China). During the experiment, food and water were freely available and the 12 h light/dark cycle was adopted. They were fed LFD (n = 6) or HFD (n = 12) for 12 weeks. After HFD feeding for 8 weeks, the mice were assigned to the HFD group (n = 6) and HFD + H_2_S group (n = 6). It has been reported that administration of NaHS (56 μM/kg/day) for 6 weeks significantly reduces serum triglyceride (TG) level, liver weight, and liver free fatty acid in HFD-fed mice (Sun et al., 2015). In this study, the mice from both HFD and LFD groups were intraperitoneally (i.p.) injected with saline and the mice from the HFD + H_2_S group were i.p. injected with NaHS (100 μM/kg/day, dissolved in saline) for additional 4 weeks. The mice were weekly weighed, as well as the food intake and water intake were determined in 24 h. After the mice were killed, the blood sample was immediately collected. In addition, the liver, inguinal/parametrial white adipose tissue, and interscapular brown adipose tissue were dissected under a Zeiss OPM 19 surgical microscope (Carl Zeiss, Oberkochen, Germany) and then weighed using an electronic scale (Mettler Toledo, Shanghai, China). Relative organ weight was calculated as the percentage of the organ weight of each group to that of the LFD group.

### Biochemical Analysis

Plasmatic levels of TG, total cholesterol (TC), aspartate aminotransferase (AST), and alanine aminotransferase (ALT) were measured by an auto hematology analyzer (Mindray, Shenzhen, China). The contents of TC and TG in liver cells, as well as the levels of TC, TG, non-esterified fatty acid (NEFA), tumor necrosis factor-α (TNF-α), interleukin (IL)-1β, and IL-6 in liver tissues were determined by ELISA kits (Jiancheng Bioengineering Institute, Nanjing, Jiangsu, China).

### Hematoxylin and Eosin and Oil Red O Staining

The liver tissues were immediately fixed in 10% neutral-buffered formalin, embedded in paraffin, sectioned at 5 μm, and stained with Hematoxylin and Eosin (HE). For ORO staining, the frozen tissues were cut into 10 μm thick sections, stained with ORO, and then counterstained with hematoxylin. The sections were observed using an Olympus BX51 microscope (Olympus, Tokyo, Japan) and analyzed by Image J software (National Institutes of Health, Bethesda, MD, United States), and the area of hepatic steatosis was determined using optical density values (Zhao et al., 2020).

### Immunohistochemistry

The sections were then stained with anti-cleaved caspase-3, anti-beclin-1, and anti-Ki67 (CST, Danvers, MA, United States) antibodies, respectively. The sections were observed using an Olympus BX51 microscope (Olympus, Tokyo, Japan). The proliferative, apoptotic and autophagic indexes were calculated by the ratios of stained cells to total number of cells.

### Statistical Analysis

The results were expressed as mean and standard error of the mean (SEM). The differences between the groups were further analyzed by one-way analysis of variance using GraphPad Prism version 6.0 followed by Tukey’s test. A *p* value <0.05 was considered statistically significant.

## Results

### H_2_S Level is Reduced in OA-Treated Liver Cells and Administration of H2S Promotes the Growth of OA-Treated Liver Cells

**FIGURE 1:**
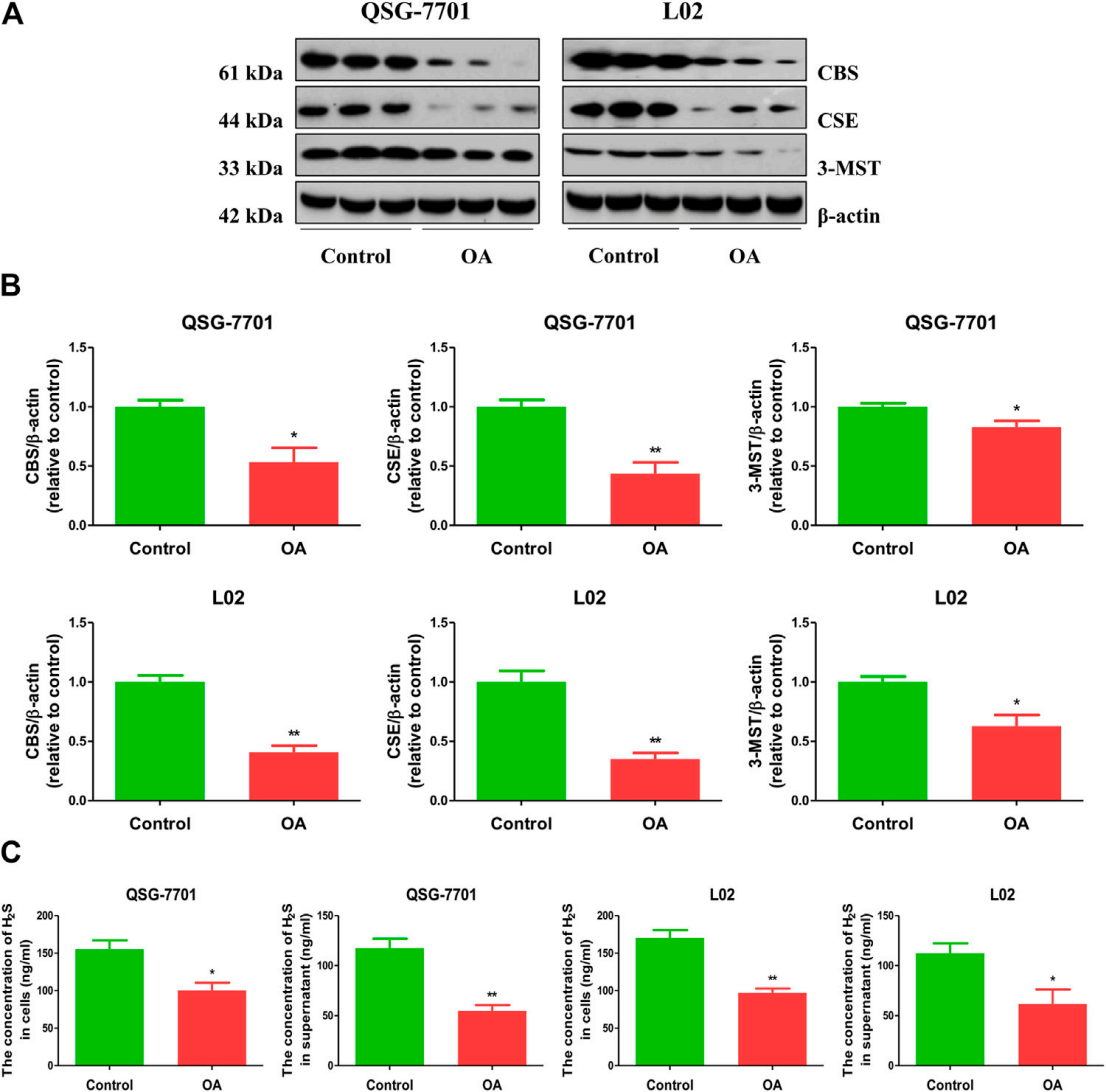
The levels of endogenous H_2_S in normal and OA-treated QSG-7701 and L02 cells and culture supernatant. **(A)** The protein levels of CSE, CBS, and 3-MST were examined by Western blot. β-actin was used as a loading control. **(B)** The densitometric quantification was performed, normalized to the level of β-actin. **(C)** The levels of H_2_S in normal and OA-treated liver cells and culture supernatant. The experiments were performed in triplicates. Data are presented as mean ± SEM; *p < 0.05, **p < 0.01 vs. control group.

**FIGURE 2:**
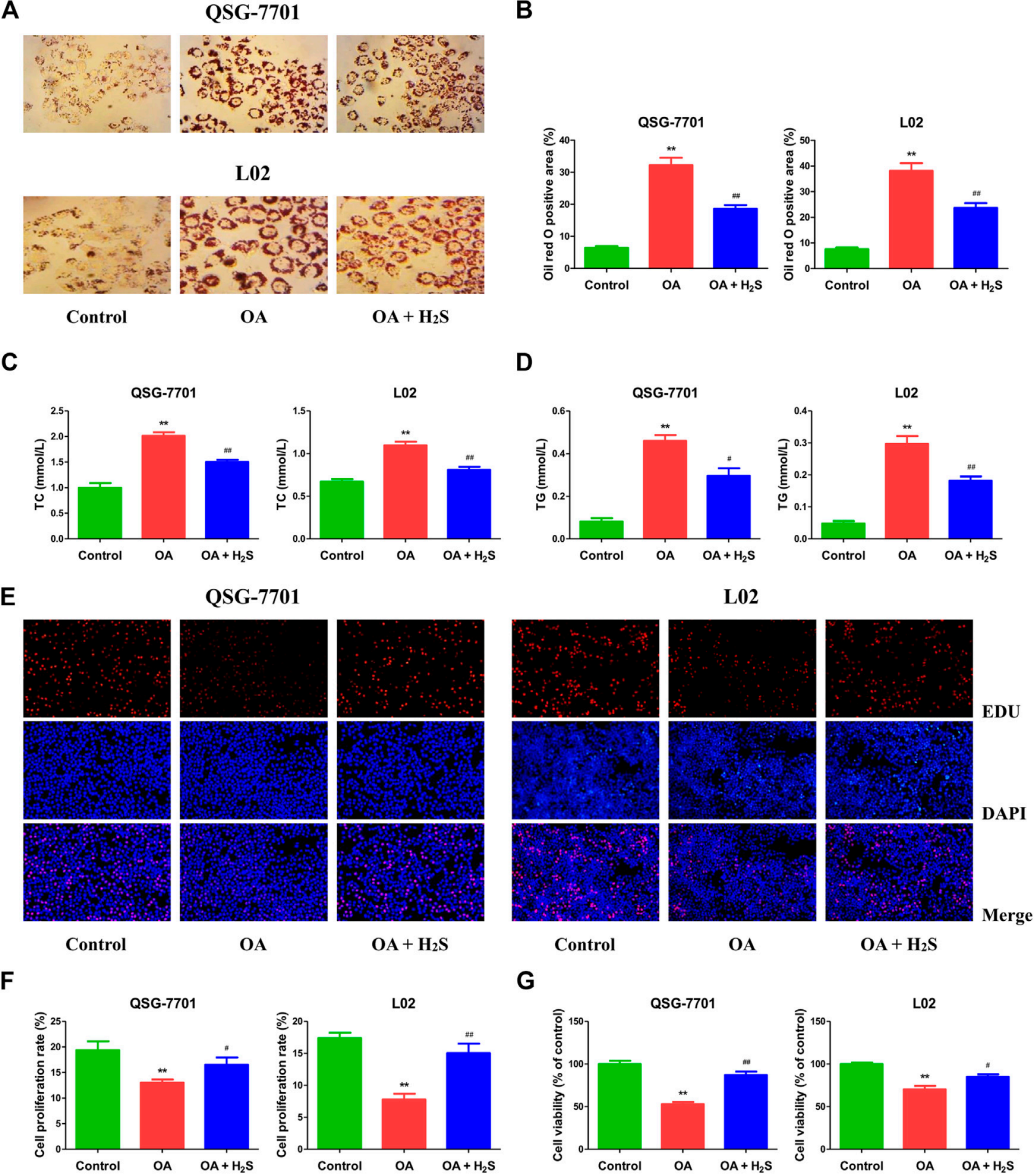
The effect of H_2_S on the formation of lipid droplet and the growth of QSG-7701 and L02 cells treated with OA. **(A)** Lipid droplets in QSG-7701 and L02 cells were stained with ORO (original magnification, ×400). **(B)** The ORO stained area was measured. **(C)** The level of TC was detected. **(D)** The level of TG was detected. **(E)** The replication activity was determined by EdU assay (original magnification, ×200). **(F)** The proliferation rate was calculated. **(G)** The percentage of viable cells was calculated. The viability of control group was normalized to 100%. The experiments were performed in triplicates. Data are presented as mean ± SEM; **p < 0.01 vs. control group; ^#^ *p* < 0.05, ^##^ *p* < 0.01 vs. OA group.

**FIGURE 3:**
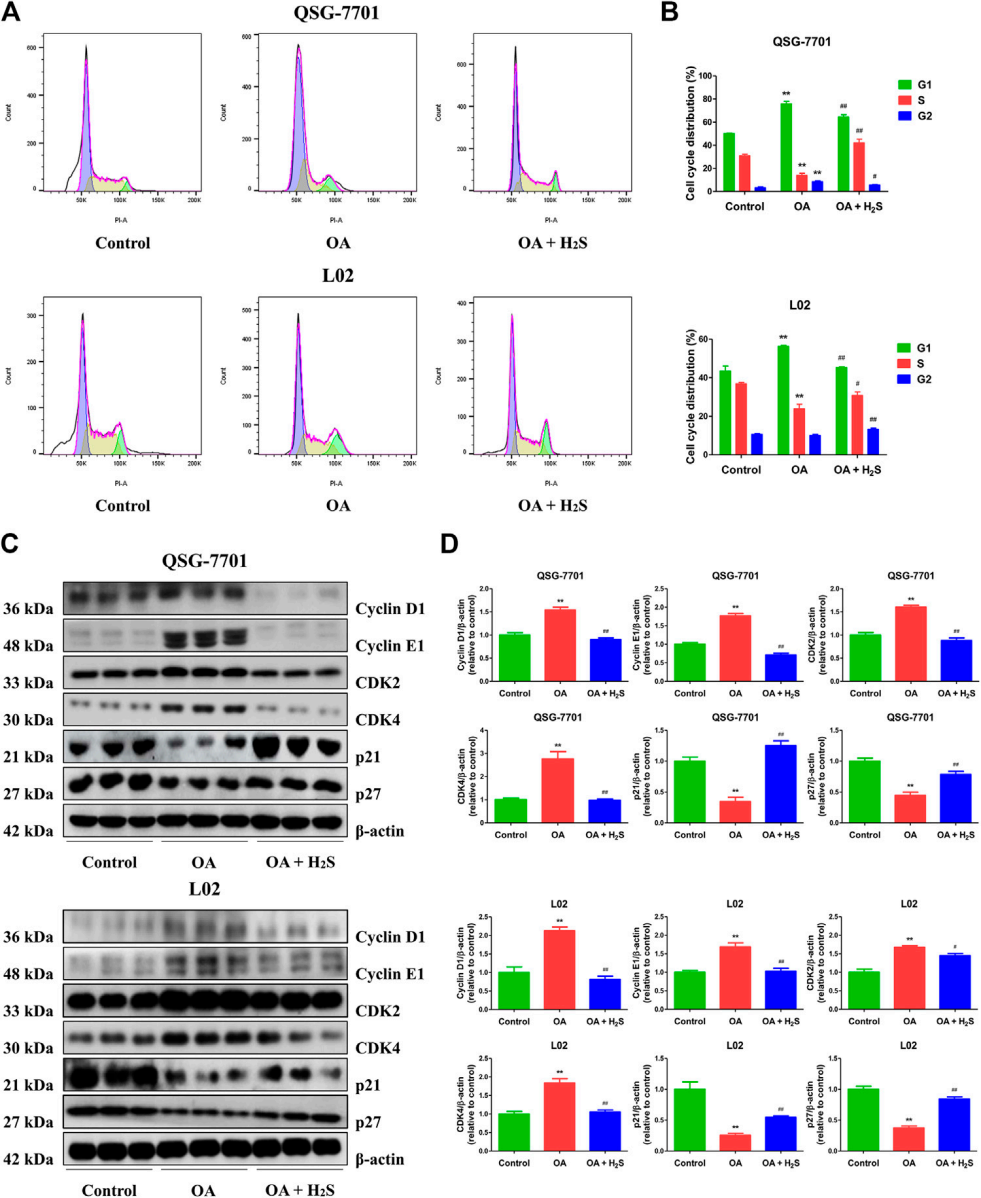
The effect of H_2_S on cell-cycle progression of QSG-7701 and L02 cells treated with OA. **(A)** The cell-cycle distribution was analyzed by flow cytometry. **(B)** Cell-cycle distribution was determined. **(C)** The expression levels of cyclin D1/E1, CDK2/4, and p21/p27 were detected by Western blot. β-actin was used as a loading control. **(D)** The densitometric quantification was performed, normalized to the level of β-actin. The experiments were performed in triplicates. Data are presented as mean ± SEM; **p < 0.01 vs. control group; ^#^ *p* < 0.05, ^##^ *p* < 0.01 vs. OA group.

The expression levels of CBS, CSE, and 3-MST in OA-treated liver cells were lower than those in control cells (Figures 1A,B). Furthermore, the concentrations of H_2_S in OA-treated liver cells and the supernatant were lower than those in untreated groups (Figure 1C). The data suggest that H_2_S is involved in the growth of liver cells. Therefore, we further detected the effects of exogenous H_2_S on the growth of cells in OA group. Our data indicated that H_2_S reduced the lipid level and the contents of TC and TG in the cells of OA group (Figures 2A–D). The proliferation and viability of liver cells were inhibited by the administration of OA, whereas H_2_S increased the proliferation and viability of the cells of OA group (Figures 2E–G). Furthermore, OA induced cell-cycle arrest during the G1-phase and H_2_S reversed the trend (Figures 3A,B). A number of cell-cycle-related proteins have been found, such as cell-cycle regulatory regulators (e.g., CDK2/4 and Cyclin D1/E1) and inhibitory cell-cycle proteins (e.g., p21/p27) (Shen et al., 2018). The data showed that OA increased the expression levels of Cyclin D1/E1 and CDK2/4, but decreased the expression levels of p21/p27, whereas treatment with H_2_S showed reverse trends (Figures 3C,D). These results suggest that H_2_S could enhance the growth of OA-treated liver cells by affecting cell-cycle progression.

### H_2_S Decreases Apoptosis in OA-Treated Liver Cells

**FIGURE 4:**
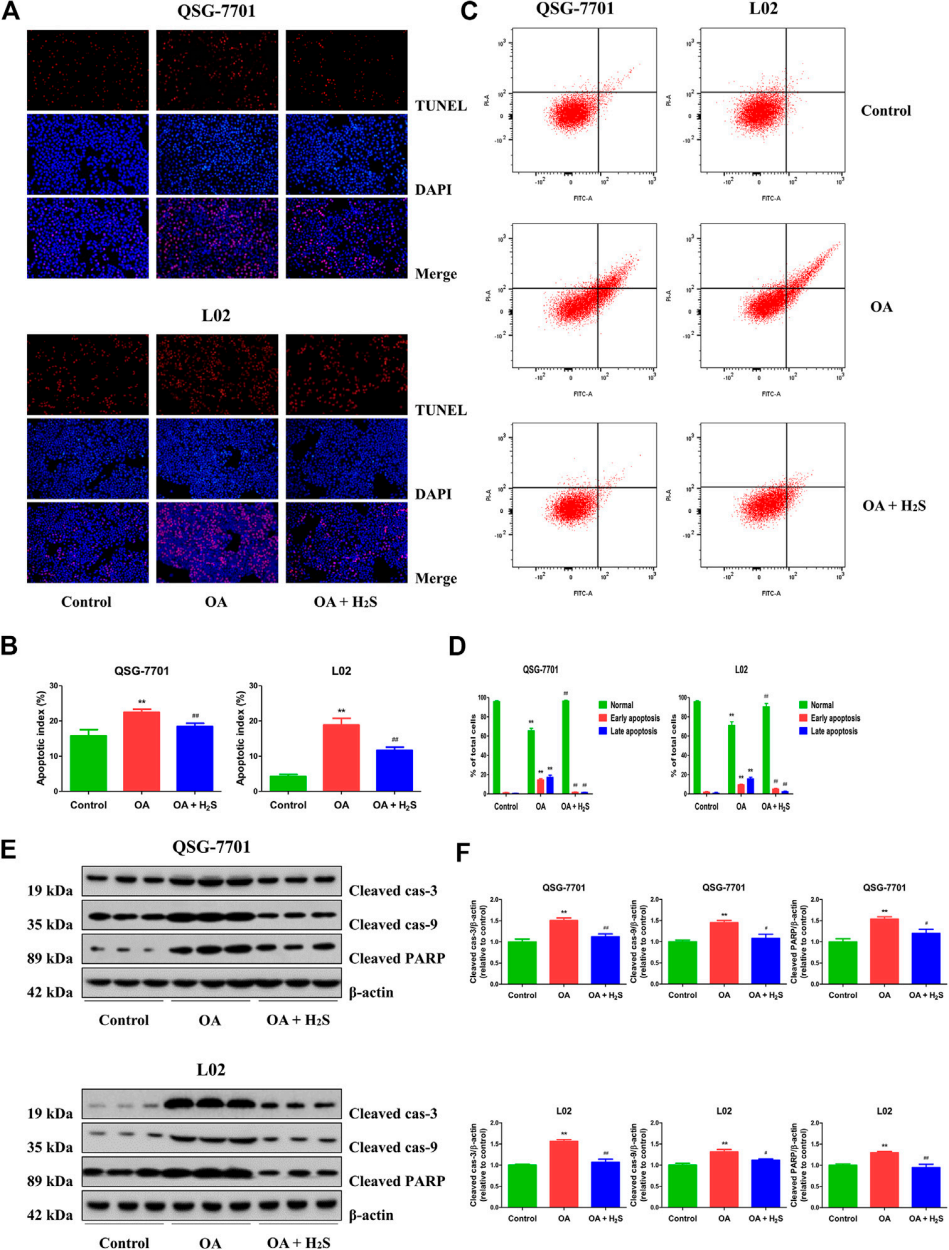
The effect of H_2_S on the apoptosis of QSG-7701 and L02 cells treated with OA. **(A)** The apoptotic levels were measured by TUNEL staining (original magnification, ×200). **(B)** The apoptotic index was calculated. **(C)** The apoptotic level was detected by flow cytometry. **(D)** The result of flow cytometry was determined. **(E)** The expression levels of cleaved cas-3, -9, and cleaved PARP were detected by Western blot. β-actin was used as a loading control. **(F)** The densitometric quantification was performed, normalized to the level of β-actin. The experiments were performed in triplicates. Data are presented as mean ± SEM; **p < 0.01 vs. control group; ^#^ *p* < 0.05, ^##^ *p* < 0.01 vs. OA group.

The apoptosis was increased in the OA group compared to the control group. Treatment with H_2_S downregulated the apoptosis in OA group (Figures 4A,B). Additionally, flow cytometry assay further demonstrated that OA can induce early/late apoptosis in liver cells, while H_2_S reduced early/late apoptosis in the cells of OA group (Figures 4C,D). Caspase family plays key roles in the process of apoptosis. Cas-3 acts as a major executioner of apoptotic cell death. Cas-9 has been considered one of the initiator caspases as they are coupled to many pro-apoptotic signals. Cleaved cas-3 and -9 can lead to the mitochondria-dependent apoptosis pathway (Man and Kanneganti, 2016). PARP plays an important role in DNA repair and acts as a target of caspases in apoptotic procession (Wu et al., 2019c). As shown in Figures 4E,F, compared with the control group, the expression levels of cleaved cas-3, -9, and cleaved PARP in OA group were higher, however the expression levels were lower in OA + H_2_S group than those in the OA group. These data reveal that apoptosis is upregulated in OA-treated liver cells and administration of H_2_S can downregulate the apoptotic level.

### H_2_S Promotes Autophagy in OA-Treated Liver Cells

**FIGURE 5:**
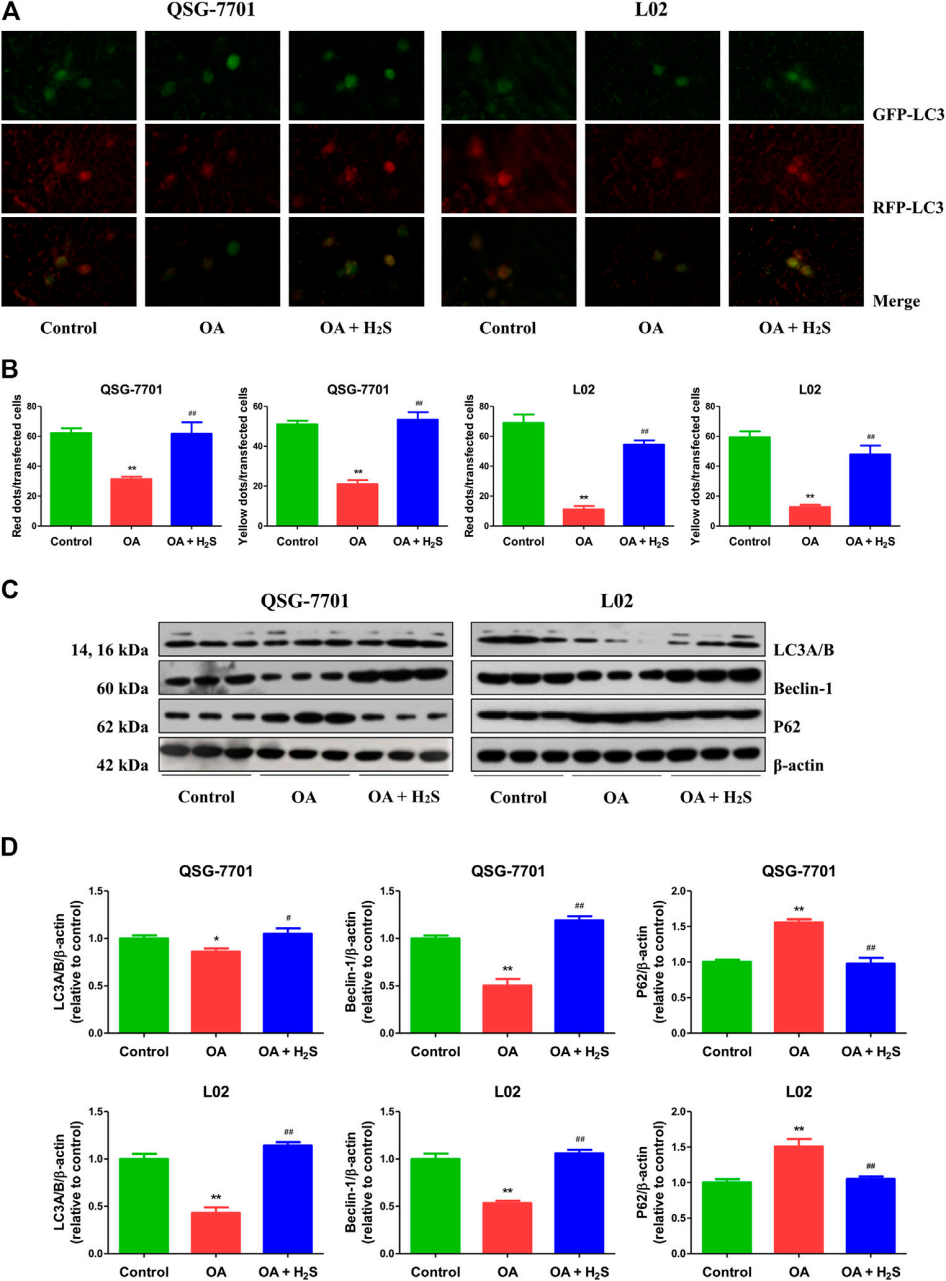
The effect of H_2_S on the autophagy of QSG-7701 and L02 cells treated with OA. **(A)** The fluorescence microscopy was used to detect QSG-7701 and L02 cells transfected with GFP-RFP-LC3 plasmid (original magnification, ×1,000). **(B)** The ratios of red/yellow dots to the transfected cells were determined. **(C)** The expression levels of LC3A/B, P62, and beclin-1 were detected by Western blot. β-actin was used as a loading control. **(D)** The densitometric quantification was performed, normalized to the level of β-actin. The experiments were performed in triplicates. Data are presented as mean ± SEM; *p < 0.05, **p < 0.01 vs. control group; ^#^ *p* < 0.05, ^##^ *p* < 0.01 vs. OA group.

Autophagy plays a crucial role in cellular homeostasis, physiology, and development through the disposal and recycling of cellular components (Wu et al., 2018). LC3 is considered a key factor of autophagy, and detection of LC3I to LC3II conversion is an important marker for autophagosome formation (Lee et al., 2017). Therefore, the GFP-RFP-LC3 plasmid was transfected into QSG-7701 and L02 cells. The numbers of yellow dots (autophagosomes) and free red dots (autolysosomes) were decreased in OA group. However, treatment with H_2_S exhibited reverse effects (Figures 5A,B). In addition to LC3, p62 and beclin 1 have been regarded as specific markers in the process of autophagy (Wu et al., 2019). Both LC3 and beclin 1 in the OA group were downregulated when compared to those of the control group, while the protein levels were upregulated in the OA + H_2_S group when compared to those of the OA group. Moreover, the protein levels of p62 showed reverse trends (Figures 5C,D). The data indicate that autophagy is reduced in OA-treated liver cells and administration of H_2_S can increase the autophagic level.

### H_2_S Inhibits ROS/PI3K/AKT/mTOR Pathway in OA-Treated Liver Cells

**FIGURE 6:**
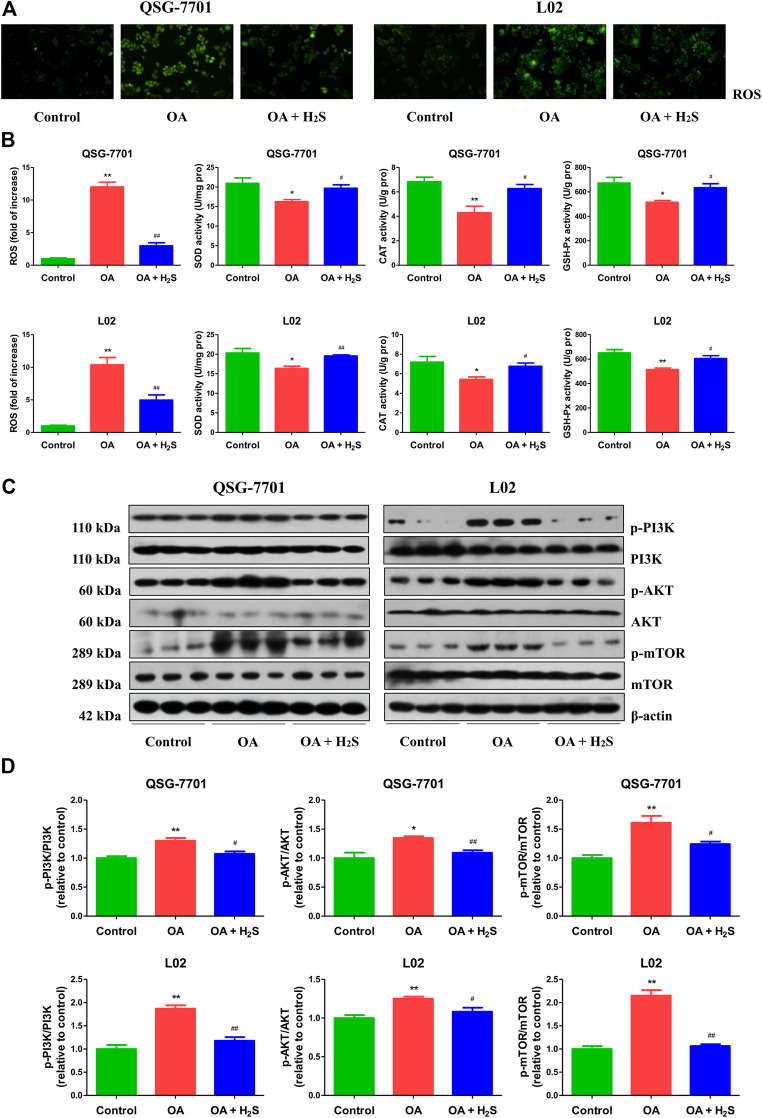
The effect of H_2_S on ROS/PI3K/AKT/mTOR pathway in QSG-7701 and L02 cells treated with OA. **(A)** The ROS level was determined (original magnification, ×400). **(B)** The ROS levels and activities of SOD, CAT, and GSH-Px were detected. **(C)** The expression levels of PI3K, p-PI3K, AKT, p-AKT, mTOR, and p-mTOR were detected by Western blot. β-actin was used as a loading control. **(D)** The densitometric quantification was performed, normalized to the level of β-actin. The experiments were performed in triplicates. Data are presented as mean ± SEM; *p < 0.05, **p < 0.01 vs. control group; ^#^ *p* < 0.05, ^##^ *p* < 0.01 vs. OA group.

ROS are chemically non-radical molecules or reactive radicals derived from molecular oxygen, such as hydrogen peroxide and superoxide anion radical (Zhou et al., 2016). SOD, GSH-Px, and CAT are crucial ROS-scavenging enzymes in mammalian cells (Zhang et al., 2018a). As can be seen in Figures 6A,B, compared to the control group, the levels of ROS were increased, but the activities of SOD, CAT, and GSH-Px were decreased in OA group, which were reversed by treatment with H_2_S. The findings indicate that H_2_S can abate OA-mediated oxidative stress in the liver. ROS are signaling messengers produced during a range of environmental stresses and have been shown to mediate the PI3K/AKT/mTOR pathway (Chen et al., 2017). We found that OA increased the phosphorylations of PI3K, AKT, and mTOR, whereas H_2_S reduced the phosphorylation levels of the proteins (Figures 6C,D). Overall, these data suggest that H_2_S could inhibit the ROS/PI3K/AKT/mTOR cascade in OA-treated liver cells.

### H_2_S Relieves NAFLD in HFD-Fed Mice

**FIGURE 7:**
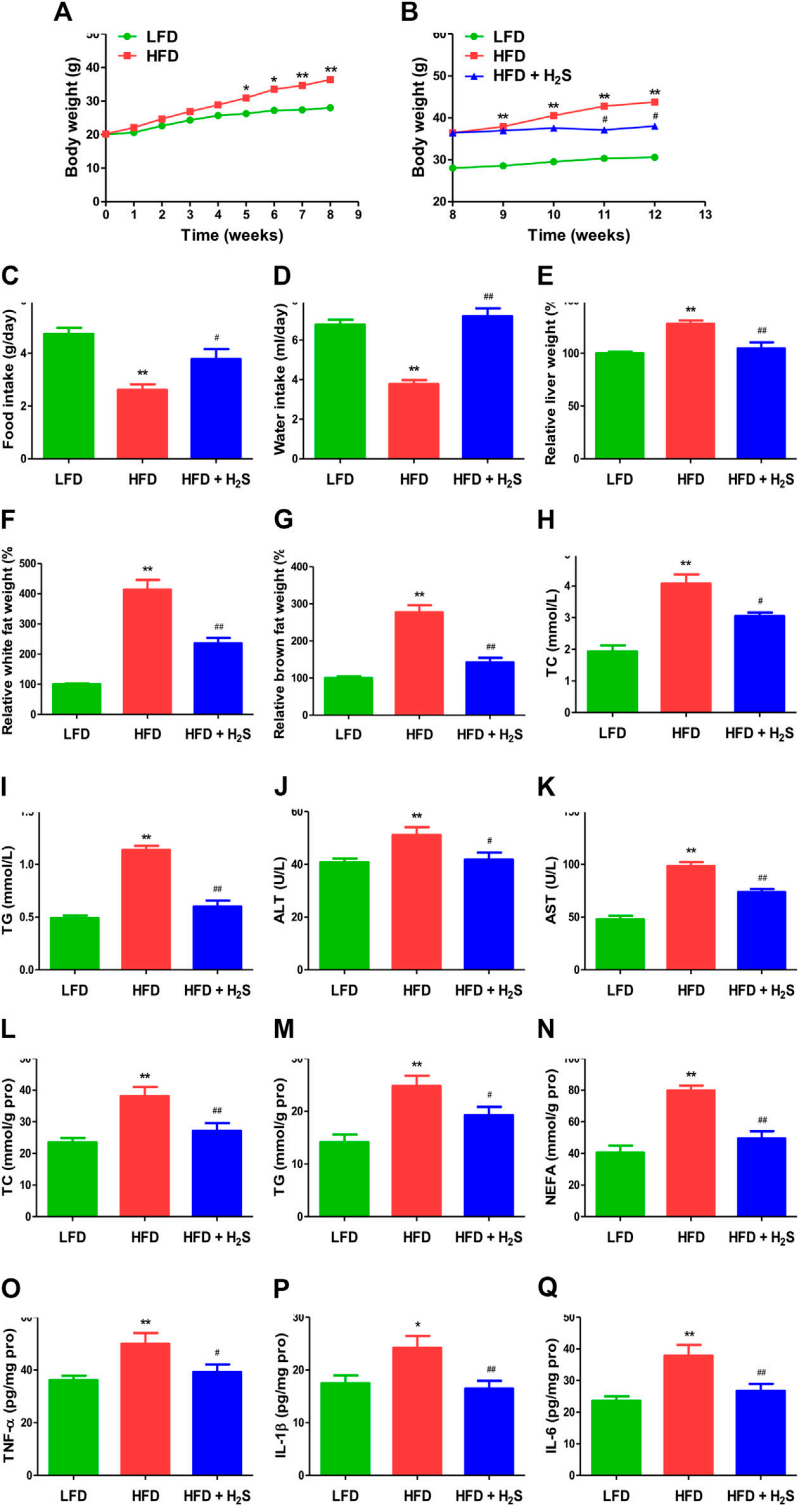
The effect of H_2_S on NAFLD in HFD-fed mice. **(A,B)** The body weight was determined. **(C,D)** Food/water intake was measured. **(E–G)** Relative liver weight and relative white/brown fat weight were determined. **(H–K)** The plasmatic levels of TC, TG, ALT, and AST were measured. **(L–Q)** The levels of TC, TG, NEFA, TNF-α, IL-1β, and IL-6 were measured in the liver of mice. Data are presented as mean ± SEM (*n* = 6). *p < 0.05, **p < 0.01 vs. control group; ^#^ *p* < 0.05, ^##^ *p* < 0.01 vs. OA group.

**FIGURE 8:**
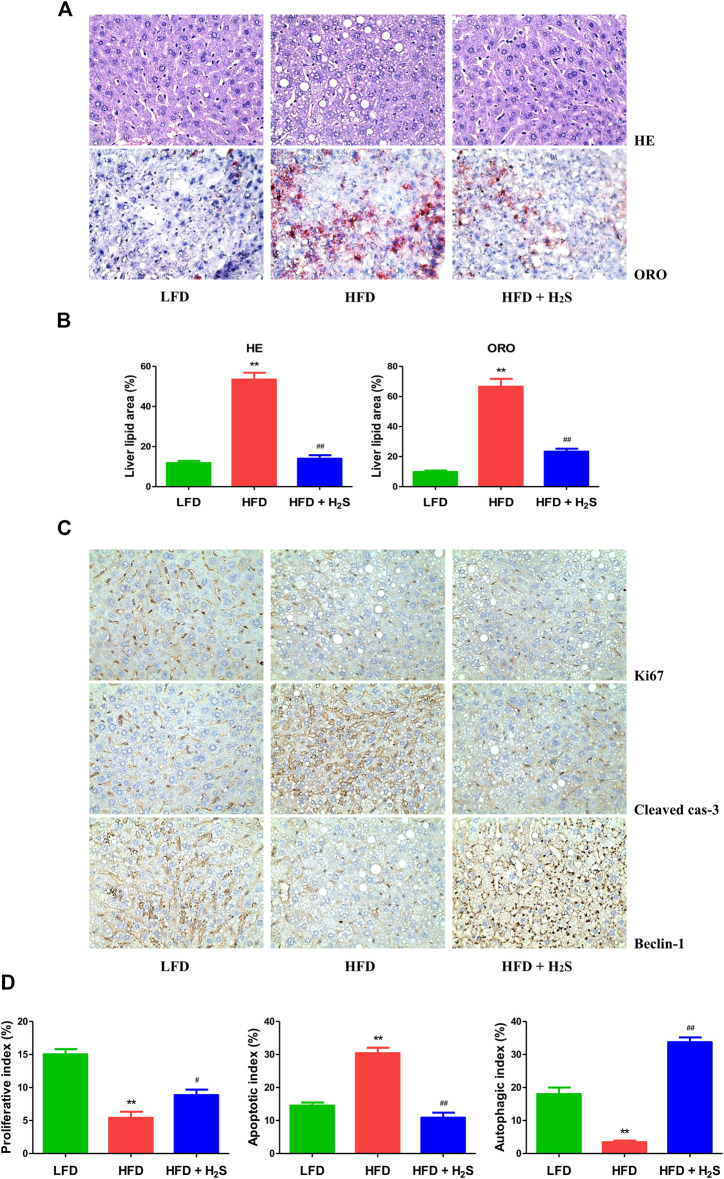
The effect of H_2_S on the proliferative, apoptotic, and autophagic activities in the liver of NAFLD mice. **(A)** Representive images stained with HE and ORO (original magnification, ×400). HFD induced vacuolar degeneration of hepatocytes, disruption of normal hepatic lobules, and inflammatory cells infiltration. H_2_S dramatically reduced hepatic lipid droplets and macrovesicular steatosis. **(B)** Liver lipid area was calculated in HE and ORO staining. **(C)** Representive images stained with Ki67, cleaved cas-3, and beclin-1 (original magnification, ×400). Ki67 and beclin-1 staining were reduced in HFD group, which were reversed by the administration of H_2_S. The results of cleaved cas-3 staining exhibited opposite trends. **(D)** The indexes of proliferation, apoptosis and autophagy were determined. Data are presented as mean ± SEM (*n* = 6). **p < 0.01 vs. control group; ^#^ *p* < 0.05, ^##^ *p* < 0.01 vs. OA group.

The body weights of HFD-fed mice were higher than those of LFD-fed mice for 8 weeks (Figures 7A,B), suggesting that the obesity model was successfully established. Food and water intakes were reduced and the relative liver, white fat, and brown fat weights were elevated in HFD-fed mice, administration of H_2_S could significantly reverse the changes (Figures 7C–G). In addition, HFD-fed mice showed elevated concentrations of TC, TG, ALT, and AST. The changes were reversed by administration of H_2_S (Figures 7H–K). HFD-fed mice exhibited increased concentrations of TC, TG, NEFA, TNF-α, IL-1β, and IL-6 in the liver, which can be reduced by H_2_S (Figures 7L–Q). The results of HE and ORO staining suggested that HFD induced vacuolar degeneration of hepatocytes, inflammatory cells infiltration, and disruption of normal hepatic lobules. Treatment with H_2_S dramatically reduced macrovesicular steatosis and hepatic lipid droplets (Figures 8A,B). Furthermore, Ki67 and beclin-1 staining were reduced in HFD group, which were reversed by H_2_S. The results of cleaved cas-3 staining exhibited opposite trends (Figure 8C). Compared with the LFD group, HFD group showed lower proliferative index and autophagic index, as well as higher apoptotic index, which were reversed by H_2_S (Figure 8D). These results together demonstrate that H_2_S could relieve NAFLD in HFD-fed mice.

## Discussion

H_2_S is one of gaseous signaling molecules and plays crucial roles in many physiological and pathological processes (Wang, 2012; Szabo, 2016; Wu et al., 2018). However, the mechanism of action of H_2_S in the development of NAFLD has not been fully investigated. Human liver cells L02 and QSG-7701 are widely used to determine the therapeutic effects of new donors/drugs (Dai et al., 2013; Wu et al., 2017a). OA, a mono-unsaturated fatty acid (FA), and palmitic acid (PA), a saturated FA, are two main dietary FAs (Liu et al., 2011). Many studies have demonstrated that OA is less apoptotic but more steatogenic than PA in hepatic cell cultures (Ricchi et al., 2009; Liu et al., 2011; Parra-Robert et al., 2019). Thus, OA was used in the current study. In the present study, L02 and QSG-7701 cells were used to detect the role of H_2_S in OA-induced NAFLD *in vitro*. Our data indicated that the expression levels of H_2_S-producing enzymes and H_2_S levels in both cells and supernatant in OA group were significantly lower than those in control group, indicating that H_2_S might play a key role in liver cell growth. It has been reported that H_2_S can increase the proliferation of neural stem cells through phosphorylation of extracellular signal-regulated protein kinases 1 and 2 (ERK1/2) (Liu et al., 2014). Furthermore, H_2_S can promote oral cancer cell proliferation by activating the cyclooxygenase 2/AKT/ERK1/2 axis (Zhang et al., 2016). We further detected the effects of exogenous H_2_S on the growth of liver cells treated with OA. The results suggested that OA reduced the proliferation and viability of liver cells, as well as induced G1-phase cell-cycle arrest, which can be reversed by treatment with H_2_S. In sum, our data suggest that H_2_S could act as an important effector molecule in enhancing the growth of liver cells treated with OA.

Apoptosis has been considered an important cell death pathway which plays crucial roles in the programmed culling of cells during eukaryotic development and organismal homeostasis (Singhet al., 2019). The underlying mechanisms of the extrinsic and intrinsic apoptotic signaling pathways have been elucidated (Ashkenazi et al., 2017). Caspases could be activated in response to a variety of apoptotic stimuli. PARP can be cleaved by caspases and activated PARP is widely adopted as a key apoptotic marker (Pang et al., 2018; Shen et al., 2019). A recent study indicates that OA can increase apoptosis via decreasing protein levels of B cell lymphoma-2 (Bcl-2) and increasing the levels of Bcl-2 associated X protein (Bax) and PARP in human hepatoma HepG2 cells (Pang et al., 2018). It has been reported that OA could induce the cleavage of cas-3 and PARP1 (Patel et al., 2016). Likewise, we found that OA could increase the apoptotic index and the protein expressions of cleaved cas-3, -9, and PARP, as well as induce early and late apoptosis in L02 and QSG-7701 cells. Administration of H_2_S decreased the apoptotic level in OA-treated liver cells. Our results indicate that apoptosis is increased in liver cells treated with OA and administration of H_2_S can decrease the apoptotic level.

Autophagy is a catabolic process that targets organelles and proteins within cells for lysosomal degradation and recycling, leading to the turnover of cellular constituents, energy production, and macromolecular synthesis (Wu et al., 2018). Autophagy can be activated by different kinds of conditions, such as hormones, nutrients, and growth factors, which can initiate cellular differentiation or exit from quiescence (Clarke and Simon, 2019). An increasing body of evidence suggests that autophagy plays a major role in adiposity and metabolic regulation (Zhang et al., 2018c). The impaired autophagic flux has been observed in lipid overloaded human hepatocytes, as well as in the liver from murine models of NAFLD and patients with NAFLD (González-Rodríguez et al., 2014; Ueno and Komatsu, 2017; Zhang, et al., 2018b). Similarly, we found that autophagy was reduced in liver cells treated with OA. A recent study has revealed that H_2_S decreases serum TG and alleviate NAFLD through the activation of hepatic autophagy via the AMP-activated protein kinase-mTOR pathway (Sun et al., 2015). Another study suggests that exogenous H_2_S can protect liver function via the induction of autophagy (Ruan et al., 2020). It has been reported that exercise training could restore bioavailability of H_2_S and promote autophagy in the liver of HFD-fed mice (Wanget al., 2017). Furthermore, aldehyde dedydrogenase-2 is involved in alleviating chronic alcohol-induced hepatic steatosis via regulation of autophagy (Guo et al., 2015). Moreover, melatonin can improve liver function in the setting of NAFLD by recovering mitophagy (Zhou et al., 2018). These results indicate that activation of hepatic autophagy may contribute to the benefit of H_2_S on HFD-induced NAFLD. In the present study, our results showed that H_2_S could increase the autophagic level in liver cells treated with OA, suggesting that autophagy activation is a promising therapeutic approach for the treatment of NAFLD. Furthermore, treatment with S-allylmercaptocysteine can ameliorate NAFLD by reducing apoptosis and enhancing autophagy (Xiao et al., 2013). Similarly, we found that H_2_S can inhibit apoptosis and promote autophagy in liver cells treated with OA. The results indicate that increased apoptotic level and decreased autophagic function are involved in the progression of NAFLD. Autophagy may modify the development of NAFLD and play a protective role in hepatocyte apoptosis (Kanda et al., 2018).

The physiological level of ROS is necessary for cell proliferation and signal transduction. However, overproduction of ROS could induce cellular redox imbalance and oxidative stress, which ultimately affect a range of cell functions (Cui et al., 2016; Wu et al., 2017b). The data suggested that OA upregulated the ROS level and downregulated the activities of SOD, CAT, and GSH-Px, which were in line with the result of a recent study (Su et al., 2018). The changes were strikingly reversed by administration of H_2_S. It has been demonstrated that the PI3K/Akt/mTOR cascade plays a role in the development of NAFLD and ROS elevation can lead to the activation of PI3K/AKT/mTOR cascade (Chen et al., 2017; Urasaki et al., 2018). A recent study indicates that scoparone could improve hepatic autophagy and inflammation in non-alcoholic steatohepatitis mice through the regulation of the ROS/p38/nuclear factor erythroid 2-related factor 2 (Nrf2) axis and PI3K/AKT/mTOR cascade in macrophages (Liu et al., 2020a). Another study suggests that S-propargyl-cysteine (SPRC, an H_2_S donor) decreases intracellular ROS levels in OA-induced HepG2 cells by upregulating AKT phosphorylation, Nrf2 translocation, and the expression levels of CSE and heme oxygenase-1 (HO-1). SPRC-induced Nrf2 translocation and HO-1 expression can be abolished by the PI3K inhibitor LY294002. In addition, the anti-oxidative effect of SPRC can be abolished by HO-1 siRNA and CSE inhibitor dl-propargylglycine. Thus, SPRC could exert the anti-oxidative effect on NAFLD via the PI3K/AKT/Nrf2/HO-1 pathway (Li et al., 2016). Similar to these findings, we observed that OA increased the expressions of p-PI3K, p-AKT, and p-mTOR. In contrast, H_2_S decreased the levels of these proteins. Furthermore, the PI3K/Akt/mTOR cascade is a crucial signaling pathway which can regulate autophagy and apoptosis (Janku et al., 2011; Vaillant et al., 2013). In sum, the data indicate that H_2_S can decrease apoptosis and increase autophagy via ROS/PI3K/AKT/mTOR cascade in liver cells treated with OA.

In the present study, an HFD-induced mouse NAFLD model was adopted and the results showed that HFD caused dramatic increases in body weight, relative liver weight, relative white/brown fat weight, and the concentrations of TG, TC, AST, and ALT in mice plasma, which suggested that the successful establishment of an NAFLD model. Administration of H_2_S significantly down-regulated the trends of these factors. It has been reported that H_2_S plays an important role in adipogenesis in white adipose tissue (Yang et al., 2018), suggesting the possibility that the lower body weight in HFD + H_2_S group may be due to less fat tissue. Many studies have shown that H_2_S can stimulate lipid formation in fat tissue but decrease lipid synthesis in the liver, indicating that H_2_S may be involved in lipid secretion from hepatocytes (Mani et al., 2015; Yang et al., 2018; Ali et al., 2020). A recent study has revealed that patients with NAFLD tended to possess upregulated concentrations of TG and TC (Zheng et al., 2017). H_2_S dramatically reduced the concentrations of TG and TC in the liver and plasma of NAFLD mice. However, another study indicates that there are no significant changes in TG and free fatty acid between normal chow diet (NCD) group and NCD + NaHS group (Sun et al., 2015). AST and ALT are sensitive indicators of liver injury in NAFLD (Lazo et al., 2015). Treatment with H_2_S effectively ameliorated liver damage in mice with fatty liver by decreasing the levels of ALT and AST. NEFA is delivered to hepatocytes for the synthesis of TG, leading to NAFLD occurrence (Jeong et al., 2017). Administration of H_2_S reduced NEFA level in the liver of mice fed with HFD. It has been reported that pro-inflammatory cytokines (TNF-α, IL-1β, and IL-6) play important roles in the development of NAFLD (Ji et al., 2018; Liu et al., 2020b; Zhao et al., 2020). Our results indicated that H_2_S reduced the levels of TNF-α, IL-1β, and IL-6 in the liver of HFD-fed mice, suggesting that H_2_S may relieve NAFLD partly by decreasing inflammation in the liver. Furthermore, H_2_S promoted autophagy and reduced apoptosis in the liver of mice fed with HFD. These results demonstrate that H_2_S can ameliorate NAFLD induced by HFD through the regulation of apoptosis and autophagy.

In conclusion, the results indicate that H_2_S level is decreased in OA-treated liver cells and exogenous H_2_S is capable of ameliorating NAFLD induced by HFD via promotion of autophagy and reduction of apoptosis through ROS/PI3K/AKT/mTOR pathway. Novel H_2_S-releasing donors may have therapeutic potential in the treatment of NAFLD.

## Data Availability Statement

All data analyzed in this work are available from the corresponding author on reasonable request.

## Author Contributions

DW, AJ, and YL participated in the conception and design of the experiments. PZ, YW, QZ, JL, and ZL performed the experiments and analyzed the data. DW wrote the manuscript. All authors read and approved the final manuscript.

## Conflict of Interest

The authors declare that the research was conducted in the absence of any commercial or financial relationships that could be constructed as a potential conflict of interest.
